# Parkinson’s disease-associated protein Parkin: an unusual player in cancer

**DOI:** 10.1186/s40880-018-0314-z

**Published:** 2018-06-26

**Authors:** Juan Liu, Cen Zhang, Wenwei Hu, Zhaohui Feng

**Affiliations:** 1 Department of Radiation Oncology, Rutgers Cancer Institute of New Jersey, Rutgers, State University of New Jersey, New Brunswick, NJ 08903 USA; 2 Department of Pharmacology, Rutgers Cancer Institute of New Jersey, Rutgers, State University of New Jersey, New Brunswick, NJ 08903 USA

**Keywords:** Parkin, Parkinson’s disease, Cancer, E3 ubiquitin ligase, Tumor suppressor

## Abstract

The mutation of the Parkin gene is a cause of familial Parkinson’s disease. A growing body of evidence suggests that Parkin also functions as a tumor suppressor. Parkin is an ubiquitin E3 ligase, and plays important roles in a variety of cellular processes implicated in tumorigenesis, including cell cycle, cell proliferation, apoptosis, metastasis, mitophagy and metabolic reprogramming. Here we review the role and mechanism of Parkin in cancer.

## Background

Parkinson’s disease (PD), the second most common neurodegenerative disorder after Alzheimer’s disease, affects 1–2% of the general population [1–3]. PD is characterized by the progressive loss of dopaminergic neurons in the substantia nigra [1–3]. Mutations of the Parkin gene *(PARK2)* have been linked to autosomal recessive juvenile PD (ARJPD), one of the most common familial forms of PD [3, 4]. Approximately 50% of the individuals with ARJPD carry *PARK2* mutations [3, 5]. Parkin dysfunction has also been implicated, albeit to a lesser degree, in the more common sporadic form of PD, as well as other neurodegenerative diseases, including Alzheimer’s disease and amyotrophic lateral sclerosis [2, 6, 7].

*PARK2* encodes a 465-residue protein comprising an N-terminal ubiquitin-like domain (Ubl), a cysteine-rich RING0 domain, and two C-terminal RING domains (RING1, RING2) separated by an “in-between RING” (IBR) domain. All these domains appear to be functionally important, since mutations in all domains have been identified in PD patients [3, 8]. Similar to many other proteins containing RING-domains, Parkin functions as an E3 ubiquitin ligase. It ubiquitinates various proteins to regulate a variety of cellular processes, including mitochondrial homeostasis, anti-oxidative stress and mitophagy (mitochondria-specific autophagy), and such actions are thought to explain at least partly how Parkin prevents PD [2, 6, 9, 10]. *PARK2* mutations could reduce its ability to ubiquitinate substrates such as CDCrel-1, Pael receptor, α-synuclein and synphilin-1, leading to their toxic build-up in the brain, which in turn causes PD [10–15]. Parkin regulates mitophagy to clear damaged mitochondria, thus preventing the accumulation of reactive oxygen species (ROS) and limiting oxidative damages in cells [9, 10, 16].

A growing body of evidence suggests that Parkin also functions as a tumor suppressor. In this review, we summarize recent advances on the tumor suppressive function of Parkin and its underlying mechanisms.

### Parkin is a tumor suppressor

**Fig. 1:**
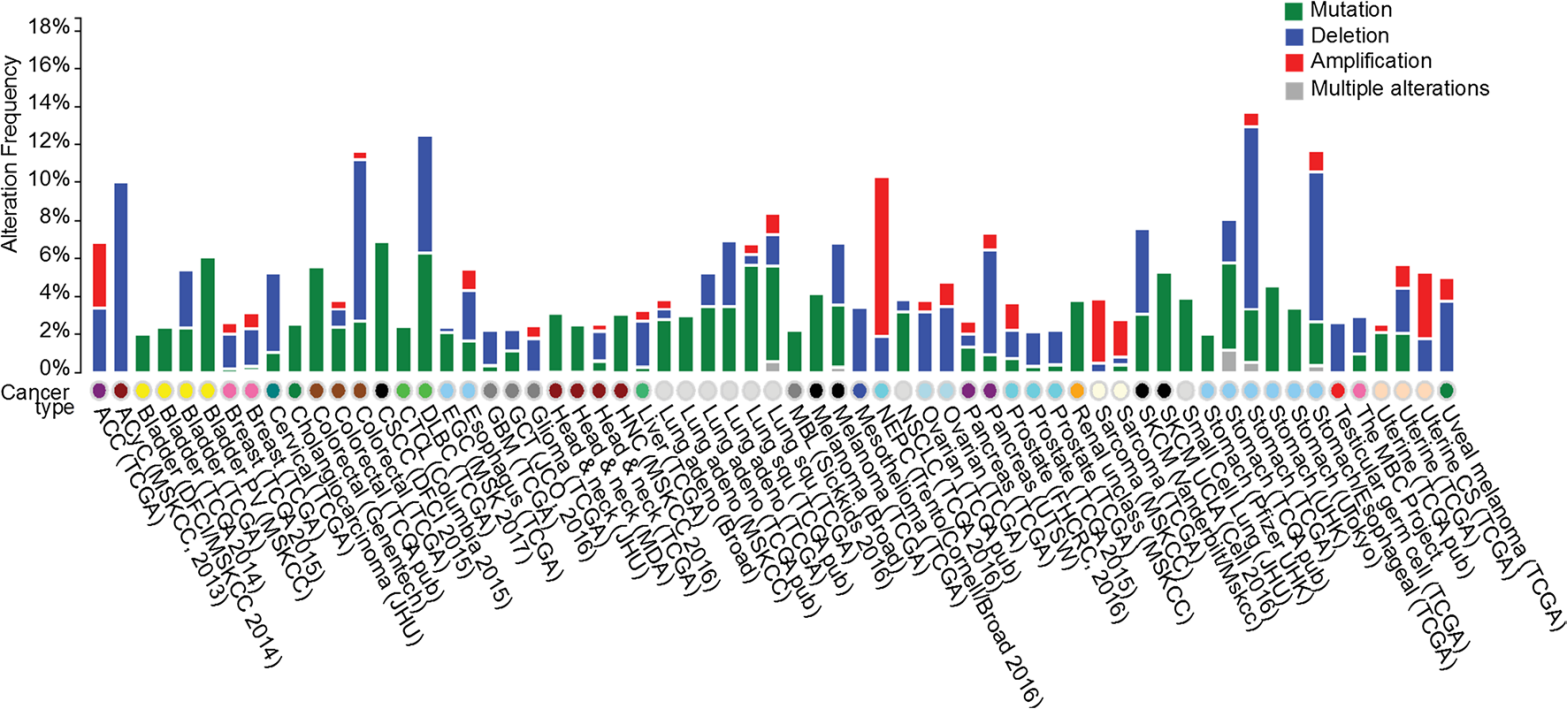
Parkin alterations in human cancers. Summary of Parkin alterations associated with different cancers, based on the datasets in cBioPortal. Deletions, mutations and amplifications are depicted in different colors

Parkin is ubiquitously expressed but predominantly in the brain. *PARK2* is localized to human chromosome 6q25-27, a region frequently lost in cancers [17]. Loss of *PARK2* heterozygosity and copy number has been observed in breast, lung, colorectal, and ovarian cancers [17–20]. Mutations of the Parkin gene have been reported in many types of cancers, although the frequency of these mutations appears to be relatively low [21, 22]. For instance, analysis of the datasets from cBioportal (http://www.cbioportal.org) [23] indicates that the Parkin gene is mutated in < 1% of breast cancer, 2–5% of colorectal cancer, ~ 5% of lung squamous cell carcinoma, and ~ 5% of gastric cancer [22, 24] (Fig. 1). Most Parkin gene mutations linked to cancer are missense mutations, with > 10% involving frameshifts or truncations [22]. Many missense mutations in cancer, such as T173A, T240M and P294S, impair E3 ubiquitin ligase activity and the tumor suppressive function of Parkin [24, 25]. In addition to mutations within the *PARK2* coding sequence, levels of *PARK2* mRNA and protein are frequently down-regulated in various types of cancers [17–20, 24]. Loss of *PARK2* heterozygosity and copy number contributes to this down-regulation; hypermethylation of the *PARK2* promoter may also be involved in certain cancers such as leukemia and colorectal cancer [26, 27].

Parkin undergoes various types of post-translational modifications that modulate its level and activity [28]. For instance, PINK1 phosphorylates Parkin to activate its E3 ubiquitin ligase activity, and recruits cytosolic Parkin to damaged mitochondria [9, 16, 29, 30]. Cyclin-dependent kinase (CDK) 5 and CK-1 phosphorylate Parkin, affecting its solubility without altering its activity [31, 32]. In contrast, c-Abl phosphorylates Parkin at Tyr 143 and inactivates it [33]. Sumoylation of Parkin promotes its nuclear localization and auto-ubiquitination [34], neddylation increases its activity [35], and *S*-nitrosylation reduces its activity [36]. In the brain, many of these post-translational modifications modulate Parkin’s neuroprotective function. It remains unclear whether these post-translational modifications are altered in cancer cells and contribute to the observed reduction in Parkin expression or activity in cancer.

Many different Parkin knockout mouse models have been established to study the role of Parkin in PD [37, 38]. However, most strains of Parkin knockout mice fail to model PD pathophysiology or display the selective loss of dopaminergic neurons characteristic of human PD [37, 38]. Instead, experiments with some of these Parkin knockout mice support the idea that Parkin is a potential tumor suppressor gene. Parkin knockout mice lacking exon 3 of *PARK2* show enhanced hepatocyte proliferation and develop macroscopic hepatic tumors that resemble human hepatocellular carcinoma, indicating that Parkin-deficient mice are susceptible to spontaneous tumorigenesis [39]. Parkin^+/−^ Apc^+/min^ mice show higher incidence of intestinal adenomas and earlier onset of all adenoma stages (monocryptal, oligocryptal, and established) than Parkin^+/+^ Apc^+/min^ mice [27]. Work from our laboratory has shown that Parkin^−/−^ mice are more susceptible than Parkin^+/+^ mice to γ-irradiation-induced tumorigenesis, although the resulting tumor spectrum (mainly lymphomas) is similar in the two strains [40].

### Mechanisms of Parkin-mediated tumor suppression

Exactly how Parkin may suppress tumor formation is poorly understood. Mechanisms proposed in the literature are discussed below.

#### Cell cycle and proliferation

**Fig. 2:**
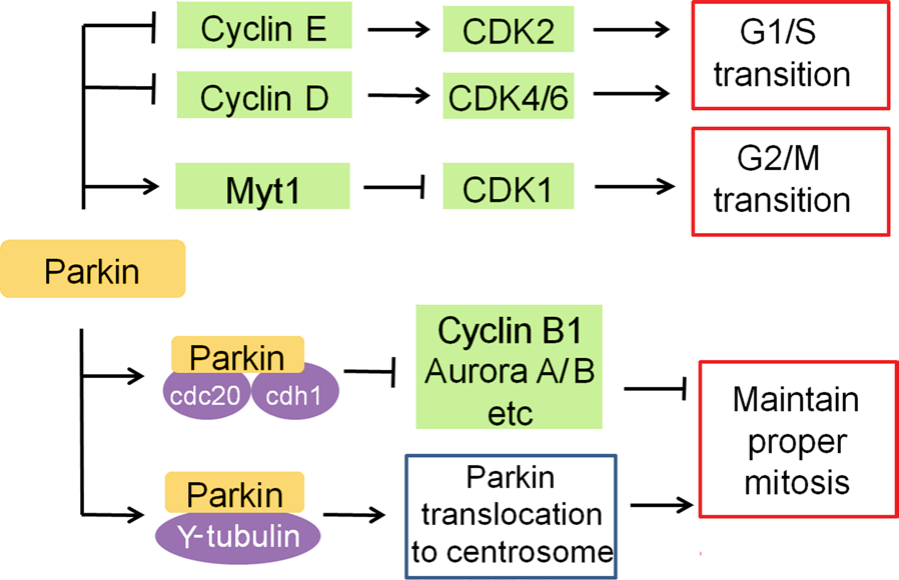
Parkin regulates cell cycle and mitosis. → : promote;  : inhibit

Parkin has been reported to play an important role in inhibiting cell cycle progression (Fig. 2). Parkin ubiquitinates and degrades cyclin E [41, 42], which in turn binds to CDK2 to promote the transition from G1 to S phase of the cell cycle. Parkin also ubiquitinates and degrades cyclin D, a regulator of CDK4/6 [43]. In this way, Parkin induces G1/S cell cycle arrest and inhibits cell proliferation. Parkin also induces expression of Myt1, which phosphorylates CDK1 to inhibit CDK1 activity and cause G2/M cell cycle arrest in HeLa cells treated with TNF-α [44].

In addition to regulating cell cycle, Parkin also regulates mitosis. Parkin interacts with anaphase-promoting complex/cyclosome (APC/C) co-activators Cdc20 and Cdh1 to mediate the degradation of several key mitotic regulators, including cyclin B1 and Aurora A/B [45]. Parkin deficiency results in overexpression of cyclin B1, Aurora A/B and other mitotic regulators, which leads to mitotic defects, genomic instability and tumorigenesis [45], suggesting that the Parkin-Cdc20/Cdh1 complex is important to ensure proper mitosis. In cells treated with the proteasome inhibitor lactacystin, Parkin forms a complex with γ-tubulin and then is recruited to the centrosome through a microtubule-dependent mechanism [46], where it may assist in mitotic spindle formation. Parkin deficiency induces spindle multipolarity and misorientation as well as multinucleation, promoting tumorigenesis [47]. These results suggest that Parkin may help suppress tumor formation by ensuring proper mitosis.

#### Apoptosis

**Fig. 3:**
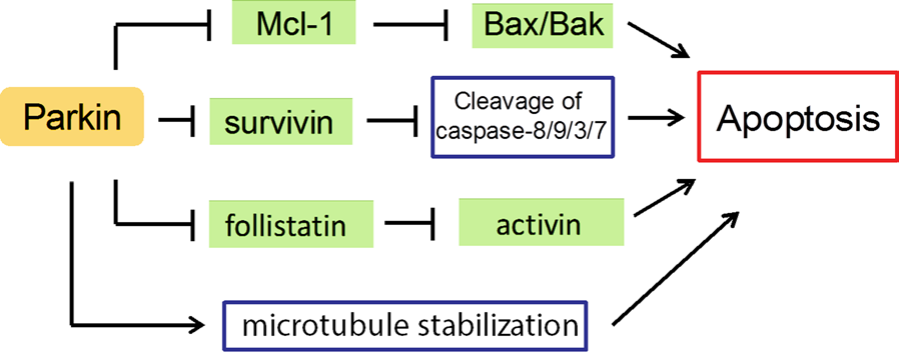
Parkin promotes apoptosis in cancer

In contrast to promoting survival of neurons [48, 49], Parkin appears to promote apoptosis of cancer cells (Fig. 3). Parkin has been reported to promote apoptosis induced by mitochondrial depolarization [50]. In response to mitochondrial depolarization caused by CCCP treatment, Parkin promotes ubiquitination and degradation of the Bcl-2 family member Mcl-1, which opens the Bax/Bak channel and thereby sensitizes cells to apoptosis. This Parkin-dependent apoptosis requires PINK1 and can be blocked by knockdown of Bax and Bak [50]. Restoration of Parkin expression in cervical cancer HeLa cells acts via a poorly understood mechanism to reduce levels of survivin, an inhibitor of apoptosis (IAP) family member that inhibits caspase, thereby sensitizing the cells to TNF-α-induced apoptosis [51]. Parkin sensitizes breast cancer MCF7 cells to apoptosis induced by microtubule-stabilizing drugs such as paclitaxel; Parkin binds to the outer surface of microtubules and increases paclitaxel–microtubule interaction [52]. Parkin also promotes apoptosis induced by HDAC inhibitors in hepatocellular carcinoma cells via a poorly understood mechanism [53]. In mouse hepatocytes, Parkin induces apoptosis by transcriptionally down-regulating follistatin, which antagonizes the pro-apoptotic cytokine activin [54, 55]. Conversely, Parkin deficiency in Parkin^−/−^ mouse hepatocytes up-regulates follistatin to inhibit apoptosis induced by chemotherapeutic agents such as cisplatin, doxorubicin and etoposide [39]. These results suggest that Parkin normally functions to keep follistatin expression at a low level and thereby limit hepatocyte proliferation, which may explain the elevated occurrence of hepatic tumor development in Parkin^−/−^ mice [39].

#### Migration, invasion and metastasis

Metastasis is a major cause of cancer-related death. Parkin expression is significantly lower in tumors with lymph node metastases than in tumors without such metastases in the case of clear-cell renal cell carcinoma [56], pancreatic cancer and nasopharyngeal carcinoma [47, 57]. Work from our laboratory has shown that Parkin is frequently down-regulated in breast cancer, and that lower Parkin expression correlates with worse distant metastasis-free survival [24]. These results suggest that Parkin plays an important role in suppressing metastasis.

**Fig. 4:**
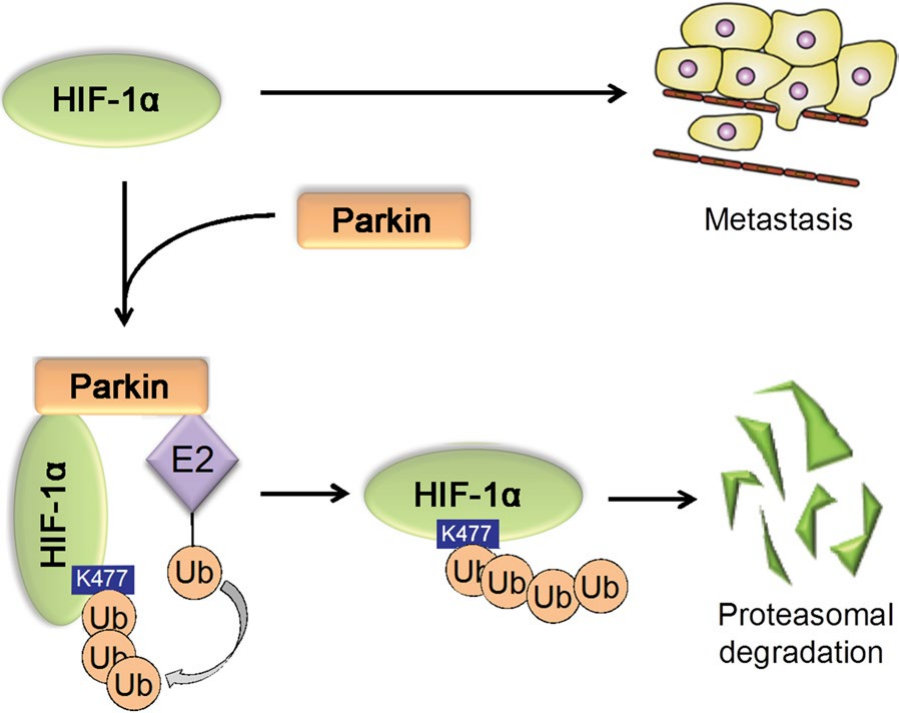
Parkin inhibits cancer metastasis by ubiquitinating HIF-1α

One mechanism through which Parkin may help suppress metastasis is by ubiquitinating HIF-1α and triggering its degradation, thereby inhibiting the migration and invasion of breast cancer cells [24] (Fig. 4). Stabilization and accumulation of HIF-1α in cancer cells promote metastasis [58, 59], and HIF-1α expression correlates inversely with Parkin expression in breast cancer specimens. Given that HIF-1α regulates, in addition to metastasis, several other cellular processes, including cell survival, metabolic reprogramming, and angiogenesis [58, 59], Parkin may exert its tumor suppressive function by inhibiting these processes, which should be addressed in future studies.

#### Metabolic reprogramming

**Fig. 5:**
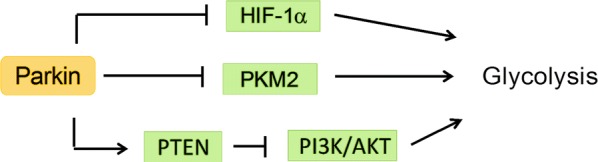
Parkin inhibits glycolysis in cancer

Metabolic reprogramming is a hallmark of cancers and plays a key role in cancer progression by driving cancer cell proliferation, survival and metastasis [60–64]. Metabolic reprogramming in cancer can be driven by the inactivation of tumor suppressors such as p53 and PTEN, or activation of oncoproteins such as HIF-1α, Myc and PI3K [60–63, 65]. The most well characterized metabolic change in cancers is enhanced glycolysis, also known as the Warburg effect [60–63, 65]. Work from our group has shown that p53, which is known to suppress glycolysis [61, 65–68], could transcriptionally activate Parkin expression by binding to the p53-responsive elements in *PARK2* [40]. Parkin suppresses glycolysis and promotes mitochondrial oxidative phosphorylation. How Parkin inhibits glycolysis is poorly understood. One possibility is that Parkin-mediated ubiquitination and degradation of HIF-1α prevents HIF-1α from transcriptionally activating the proteins involved in glycolysis [24] (Fig. 5). Similarly, another group reported that p53 transcriptionally activates Parkin in glioma [69]. The same group reported that Parkin, independently of its E3 ligase activity, transcriptionally represses p53 expression in mouse and human brains affected by ARJPD, suggesting that p53 can form a negative feedback loop with Parkin in the brain [70]. Further studies are needed to clarify whether this negative feedback loop exists in tissues outside the brain and in tumors.

Similar to p53, PTEN is involved in Parkin-mediated metabolic regulation. Parkin deficiency promotes PTEN inactivation through *S*-nitrosylation and ubiquitination, which activates PI3K/AKT signaling in cancer cells [71] (Fig. 5). PI3K/AKT signaling drives metabolic reprogramming in cancer cells, including promotion of glycolysis [60–64].

Parkin interacts with PKM2, a glycolytic enzyme that is frequently overexpressed in cancer, and catalyzes ubiquitin conjugation to PKM2 [72]. This ubiquitination inhibits PKM2 activity without destabilizing the protein. Loss of Parkin function in cancer cells enhances PKM2 enzymatic activity, thus promoting glycolysis [72] (Fig. 5).

These studies suggest that one mechanism by which Parkin suppresses tumorigenesis is by inhibiting glycolysis.

#### Mitophagy

Parkin-mediated neuroprotection involves regulation of mitophagy [9, 10, 16]. In response to mitochondrial damage, the serine/threonine kinase PINK1 is stabilized and accumulates on the outer membrane of the damaged mitochondria, where it phosphorylates Parkin and thereby activates its E3 ubiquitin ligase activity and recruits it from cytosol to damaged mitochondria [9, 16]. Parkin ubiquitinates outer mitochondrial membrane proteins, triggering mitophagy that selectively clears damaged mitochondria [9, 16]. Thus, PINK1 and Parkin work together to prevent PD by eliminating the accumulation of damaged mitochondria, ROS and mitochondrial DNA mutations [9, 10, 16].

Consistent with the idea that Parkin is important for both mitophagy regulation and tumor suppression, mitophagy defects have been linked with cancer development [73, 74]. In parallel, BNIP3 and Nix (also named BNIP3L) induce mitophagy independently of Parkin and suppress growth of certain types of tumors [10, 73, 74]. BNIP3 is silenced in liver, lung, pancreatic and colorectal cancers [75–77], and this loss leads to mitophagy defects, increases glycolysis and ROS production, and promotes growth and metastasis of breast cancer cells in mouse models [78, 79]. Similarly, knockdown of Nix promotes tumorigenesis in mouse models [80]. These studies suggest that loss of mitophagy mediated by Parkin or BNIP3/Nix contributes to the progression of certain types of cancers.

Analogously to the dual role of autophagy in cancer, mitophagy may promote or suppress tumorigenesis depending on the cellular circumstances [10, 73, 74]. Future studies should examine the role of mitophagy in the development and progression of different types of cancers.

## Conclusions and perspectives

Increasing evidence from cell culture systems, xenograft tumor models, Parkin knockout mouse models, and clinical studies suggests that Parkin is an important tumor suppressor. How the protein suppresses tumor growth is poorly understood, with many questions requiring further study. One question is how Parkin inhibits apoptosis and promotes survival in neuronal cells, yet promotes apoptosis in various types of cancer cells and mouse hepatocytes. One possible explanation is brain-specific regulation of p53 by Parkin. Induction of apoptosis is an important mechanism by which p53 exerts its tumor suppressive function in cancers [81, 82]. Interestingly, p53 activation has been observed in the brain of PD patients as well as mouse models for PD mice, suggesting that p53 activation and p53-mediated apoptosis contribute to PD [83–87]. Parkin inhibits p53 activation in the brain [70]. Accordingly, inhibition of p53-mediated apoptosis could be an important mechanism that contributes to the neuroprotective effect of Parkin. It remains unclear whether Parkin affects p53 activation or p53-mediated apoptosis in cancer cells. Future work should address this question.

Another unanswered question is why Parkin is down-regulated in many types of cancers. This cannot be explained solely on the basis of mutations, loss of heterozygosity or copy number, and promoter hypermethylation. Future studies should examine whether the transcriptional regulation of *PARK2*, epigenetic modifications of the gene, or post-translational modifications of Parkin (e.g., include phosphorylation, ubiquitination, sumoylation, neddylation, and *S*-nitrosylation) are altered in cancer [22, 28].

The third question is whether and how the anti-cancer activity of PD relates to the function of Parkin in PD. Many epidemiological studies have indicated an association between PD and reduced risk of prostate, lung, bladder, stomach, uterine, and colorectal cancers, and increased risk of melanoma, brain and breast cancers [88–91]. The association between PD and cancer risk appears to be complex and may be linked to factors such as ethnicity: PD among Asians in one study was linked to increased risk of brain, kidney, uterine, stomach and lung cancers [92]. Future studies should examine why PD shows opposite associations with different types of cancers, or even opposite associations with the same cancer in different patient populations. Future studies should also examine the potential role of Parkin gene mutations in mediating the association between PD and cancer risk. Such analyses will need to take genetic, epigenetic and environmental factors into account.

The role of Parkin in PD is well established, and its role as a tumor suppressor has recently emerged. How Parkin protects against PD and cancer is poorly understood. Future studies in this area may lead to novel therapeutic targets and strategies for both diseases.
